# Effect of different sweeteners on the oral microbiota and immune system of Sprague Dawley rats

**DOI:** 10.1186/s13568-020-01171-8

**Published:** 2021-01-06

**Authors:** Xi Cheng, Xiurong Guo, Feihong Huang, Hui Lei, Quan Zhou, Can Song

**Affiliations:** 1 Department of Stomatology, People’s Hospital of Leshan, Leshan, 614000 Sichuan China; 2 School of Pharmacy, Southwest Medical University, Luzhou, 646000 Sichuan China

**Keywords:** Oral microbiota, Oral immune system, High-throughput sequencing, Sweetener, Microbiome

## Abstract

Sucrose, xylose, and saccharin are commonly used beverage additives and long-term consumption of these compounds inevitably affects the oral immune system and the composition of oral microbiomes. In this study, we used 24 Sprague Dawley rats divided into four groups, i.e., sucrose, saccharin, xylose, or pure water treated over an eight week period to evaluate any changes in the composition, community structure, and function of the oral microbiomes. At the end of the treatment period, we collected oral microbiome samples from each animal and subjected them to high-throughput sequencing. We also used ELISA to determine the concentration of salivary immunoglobulin in these rats to reveal the effect of sweetener on the oral immune system. Sequencing results demonstrated that *Firmicutes* and *Proteobacteria*, remained the predominant phyla, but we found that the oral microbial diversity of rats drinking sucrose water was significantly higher than that of the other groups. Our results indicate that drinking water supplemented with sweeteners may influence oral immunity as well as the composition, metabolic function, and diversity of the oral microbiota, thereby disrupting the oral microbiome.

## Introduction

The oral microbiota is an essential component of human health, with changes in this microbiome potentially altering immune function, food metabolism, and colonization by potentially pathogenic microbes (Dewhirst et al. 2010). Changes in the oral microbiota may have unpredictable effects on host health. Sucrose, xylose, and saccharin are common beverage additives with these sweeteners, irrespective of their type, inevitably affecting the delicate balance within the oral microbiota (Wade 2013). Many studies have focused on the effects of sweeteners on the intestinal microbiota (Suez et al. 2014), but little attention has been paid to the oral microbiome. Oral immunoglobulins are vital components of the human oral immune system (Berglund 1971) with several oral diseases being closely associated with changes in the concentration of these proteins (Kaufman and Lamster 2000; Sistig et al. 2002; Jensen-Jarolim et al. 2008; Helfand et al. 1996). Sucrose plays a role in increasing appetite and nutrition in humans, making it a favorite additive throughout history. However, numerous studies have shown that long-term intake of excess sucrose can cause serious illness, including promoting diabetes, obesity, and caries (Khiraoui and Guedira 2018). Other sweeteners, such as xylose and saccharin, cannot be broken down by human digestive enzymes, and are seldom absorbed by the gastrointestinal tract, resulting in their direct excretion, reducing their overall impact on caloric intake (Chattopadhyay et al. 2014). This means that both xylose and saccharin are being increasingly used in beverages as alternatives to sucrose. In addition, recent studies have reported that xylose and saccharin exert a detrimental effect on the gut microbiome (Martínez-Carrillo et al. 2019; Inan-Eroglu and Ayaz 2019). Previous research into the effects of sweeteners on the human microbiota have primarily focused on the gut with relatively few studies evaluating the oral microbiome in any detail. Moreover, relatively few studies have compared the effects of sucrose and other sweeteners on the oral microbiota. Here, we aimed to address this need by focusing on the oral microbiome.

The aim of this study was to explore the effects of sucrose, xylose, and saccharin on the oral microbiota and immunity of Sprague Dawley (SD) rats. To this end, we fed SD rats with various concentrations of sucrose, xylose, and saccharin water for eight weeks. Rats were then anesthetized and saliva and oral microbiome samples were collected. These samples were then analyzed using 16S rRNA sequencing and we evaluated any differences in community structure, metabolic function, or composition of the oral microbiota in these four groups. ELISA was used to estimate the concentrations of IgG, IgE, IgM, and SIgA and we found that the oral immune system and microbiota were affected by all three substrates. Sucrose, xylose, and saccharin affect the oral micro-environment and micro-ecological balance with most of these changes being inextricably linked to the oral health of the host.

## Material and methods

### Animals and experimental design

Six-week-old female SD rats were purchased from Dashuo Co. (Chengdu, Sichuan Province, China) and maintained in a controlled environment with a temperature of 14–22 °C, humidity of 40–70%, and a 12 h light: dark cycle. The rats were randomly divided into four groups: control, sucrose, xylose, and saccharin (six rats per group) and the animals were fed ad libitum (Dashuo Company). Based on the findings of previous experiments (Uebanso et al. 2017), and using the principle of low-dose sweetener consumption, rats in each of the different groups were provided with drinking water supplemented with 0.83 mg/mL xylose, 0.83 mg/mL sucrose, or 0.83 mg/mL saccharin for 12 h/day, and purified water for the 12 h at night. After eight weeks of feeding, naturally secreted saliva was collected from the hypoglottis of these animals using a micro sampler, with 30 μL of saliva being collected from each rat and stored in a PE pipe. All rats were anesthetized using an intraperitoneal injection of 3% Pentobarbital Sodium, and microbiome samples were collected using a small sterile cotton swab gently rotated under the tongue, palate, and upper throat of the rat’s oral cavity. These cotton swabs were then snapped and the head was placed into a sterile PE tube. All samples were incubated on dry ice under sterile conditions until they could be shipped for evaluation.

### ELISA

Enzyme-linked immunosorbent assay (ELISA) was used to evaluate the SIgA, IgE, IgM, and IgG levels in the rat saliva samples according to the manufacturer’s protocol (Tengfei Inc., Shenzhen, China). Four 24-well culture plates were used for the four groups of saliva samples (Tengfei Inc.). Each experiment was repeated three times, and the saliva samples from the control, xylose, saccharin, and sucrose groups were added to the wells at a volume of 10 µL/well and diluted with 40 µL of diluent. Next, we added 100 μL of horseradish peroxidase (HRP)-conjugated primary antibody to the wells and incubated the plates at 37 °C for 60 min. The supernatant was then discarded, the cells were washed and dried five times before the 3,3′,5,5′-tetramethylbenzidine (TMB) substrate was added to each well and incubated at 37 °C in the dark for 15 min. The reaction was terminated by adding 50 µL of the stop solution to each well and the concentration was evaluated using a microplate reader.

### DNA isolation, PCR amplification, and MiSeq analysis

Microbial DNA was isolated from the oral swab samples using the E.Z.N.A@soil DNA Kit (Omega Bio-Tek, Norcross GA, USA) according to the manufacturer’s instructions. A NanoDrop 2000 UV–vis spectrophotometer (Thermo Scientific, USA) was used to determine the final concentration and purity of DNA and a microbial DNA quality check was completed using a 1% agarose gel. The V3–V4 hypervariable regions of the bacterial 16S rRNA gene can be amplified using a thermocycler PCR and specific primers 338F (5′-ACTCCTACGGGAGGCAGCAG-3′) and 806R (5′-GGACTACHVGGGTWTCTAAT-3′). PCR amplification was then completed as follows: initial denaturation at 95 °C for 3 min; denaturation at 95 °C for 30 s (27 cycles), annealing at 55 °C for 30 s, extension at 72 °C for 45 s (27 cycles), and final extension at 72 °C for 10 min. The total volume of the PCR was 20 µL and consisted of the following: 0.4 µL FastPfu DNA polymerase (TransGen Biotech Co., Beijing, China), 2.5 mM dNTPs (2 µL), template DNA (10 ng), 5 µM of each primer (0.8 µL), and 5 × FastPfu buffer (4 µL). PCR products were separated on 2% agarose gels and then purified using an AxyPrep DNA gel extraction kit (Axygen Biosciences, Union City, CA, USA). Finally, the purified PCR products were quantified using a QuantiFluor-ST (Promega, USA) and pooled to produce equimolar concentrations and sent for paired-end sequencing (2 × 300) on an Illumina MiSeq platform (Illumina, San Diego, USA) according to the standard protocol described by Majorbio Bio-Pharm Technology Co. Ltd. (Shanghai, China) and all data were deposited on NCBI (Accession Number: PRJNA607824). Before analysis, sequences were demultiplexed, quality-filtered, and subsampled using the FLASH (version 1.2.11) and Qiime (version 1.9.1) software. Operational taxonomic units (OTUs) were clustered using UPARSE software (version 7.1 http://drive5.com/uparse/) with a 97% similarity cut off, and chimeric sequences were identified and removed using UCHIME. Taxonomic analysis of each sequence was then completed using the RDP Classifier algorithm (version 11.5, http://rdp.cme.msu.edu/) and the Silva (SSU123) 16S rRNA database at a confidence threshold of 70%.

### Ecological and statistical analyses

We used cloud platform (http://www.majorbio.com/) to draw a sparse curve for each sample in order to accurately identify and describe the microbiota within each of our experimental groups. Sequencing depth (coverage), sobs, Simpson, Shannon–Wiener, and alpha diversity indices were all calculated using mothur software (version 1.30.2) and used to describe the microbial diversity of the samples. The principal coordinate and Adonis analysis of the microbial composition of each sample was calculated using the OTU values and plotted using the R (programming language) software package (version 3.3.2). Venn diagrams were used to reveal unique and shared groups within the microbiota of the experimental animals and were drawn using the R software package. In order to evaluate the significance and biological relevance of this data linear discriminant analysis effect size (LEfSe) was used to identify differentially expressed OTUs between the four treatment groups. A Kruskal–Wallis test, FDR correction, and Scheffe test were used to analyze the differences among multiple groups, and a student’s *t*-test, two-tailed test and FDR correction were used to analyze the differences between two groups. A *p* value of < 0.05 was considered significant.

### Functional prediction

We used PICRUSt (phylogenetic investigation of communities by reconstruction of unobserved States) to predict the functional features of the microbiomes in each sample. PICRUSt was accessed via the Majorbio free cloud platform (www.majorbio.com) and used the KEGG (Kyoto Encyclopedia of Genes and Genomes) database for functional predictions. The abundance of specific functions within each group were calculated. These results were then reported using a box diagram of the COG functional classification statistics.

## Results

### ELISA

**Fig. 1:**
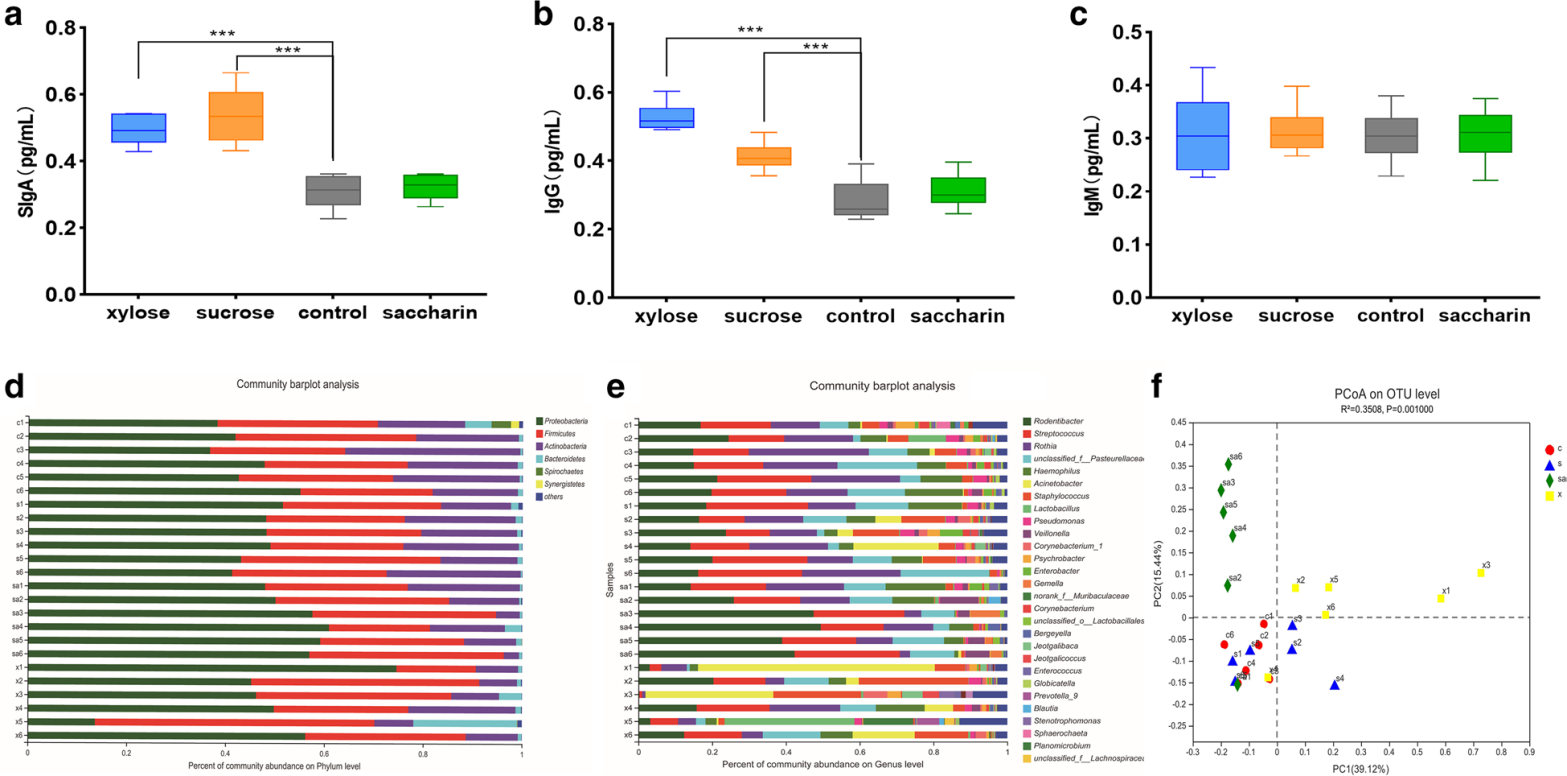
This image consists of six parts, **a**, **b**, and **c** representing the results of each of the individual rat saliva immunoglobulin ELISAs. Oral bacterial community at the phylum (**d**) and genus (**e**) levels. Relative abundance of bacterial groups in each of the 24 oral cavity samples (black). Any phylum or genus with an abundance of less than 1% was merged into other groups. **f** Principal coordinate analysis (PCoA) of the microbial communities. Red circles, Blue triangles, Green diamonds, and Yellow squares represent the gut microbiotas of samples from the control (c group), sucrose (s group), saccharin (sa group), and xylose (x group) groups, respectively. * 0.01 < *p* ≤ 0.05, ** 0.001 < *p* ≤ 0.01, *** *p* ≤ 0.001

Immunoglobulins are a critical class of immune-activators known to facilitate proper immune response. Oral immunoglobulins include varying concentrations of SIgA, IgG, IgM, IgD, and IgE. Here, we used an ELISA against rat SIgA, IgG, IgM, and IgE in rat saliva, to evaluate the changes in immunoglobulin expression following exposure to different sweeteners. The ELISA results are summarized in Fig. 1a, b, and c. IgA, IgM, and IgG were detected in all of the samples while IgE was not detected in any of them. The mean concentrations of SIgA and IgG were significantly higher in the xylose and sucrose groups when compared to those in the control (Fig. 1a and b) but no significant differences were observed in SIgA and IgG concentrations between the saccharin and control groups (Fig. 1a and b). In addition, there were no significant differences between the IgM concentrations of any of the groups (Fig. 1c).

### Taxonomic composition and Community structures

After denoising the sequencing data from each of the 24 samples, we were able to identify, on average, 39,987 sequences per sample. The Rarefaction curves, Shannon curves, Simpson curves, Coverage curves, and diversity indices of each sample demonstrated a genetic distance of 3% between groups and are summarized in the Additional file 1 (see Figures S1–S4, and Table S1). Our sequencing data could be divided into 1057 OTUs, with the number of OTUs per sample ranging from 410 to 739. Only the *Firmicutes* and *Proteobacteria* were identified in all 24 saliva samples, with *Proteobacteria* accounting for 36.71–60.91% of the 16S rRNA gene sequences (Fig. 1d). Firmicutes were found to contribute 20.42–56.53% of the total reads per sample (Fig. 1d). When these data were evaluated at the genus level, the top four abundant genera in each group were listed in Table 1. Based on the bacterial genus data, detected OTUs were distributed 496 different genera (Fig. 1e). Principal coordinate analysis (PCoA) was used to evaluate the community structure of the rat oral microbiota. In Fig. 1f, each symbol represents a specific sample while the red, green, yellow, and blue dots represent the control, saccharin, xylose, and sucrose groups, respectively. Figure 1f shows that the bacterial communities in the control and sucrose groups were tightly clustered on the principal coordinate and separated from the oral bacterial communities in the xylose and saccharin groups along the central parallel axis 1 (PC1). This result was shown to be the most significant variable (35.25%). The Adonis analysis revealed that these changes represent significant differences in the community structure of these sample groups (R^2^ = 0.3508, *p* = 0.001, Fig. 1f).

**Table 1:** The top 4 abundance bacteria genus among the four groups of rats oral microbiota

Sample group	Top four abundant genera	Composition ratio (%)
Sucrose group	*Streptococcus*	20.25
	*Rodentibacter*	18.34
	*Rothia*	16.81
	*Pasteurellaceae*	10.82
Xylose group	*Acinetobacter*	19.56
	*Staphylococcus*	13.20
	*Streptococcus*	8.92
	*Rodentibacter*	6.85
Control group	*Rothia*	20.47
	*Streptococcus*	19.11
	*Rodentibacter*	18.84
	*Pasteurellaceae*	10.69
Saccharin group	*Rodentibacter*	36.05
	*Streptococcus*	21.70
	*Rothia*	10.73
	*Pasteurellaceae*	10.54

**Fig. 2:**
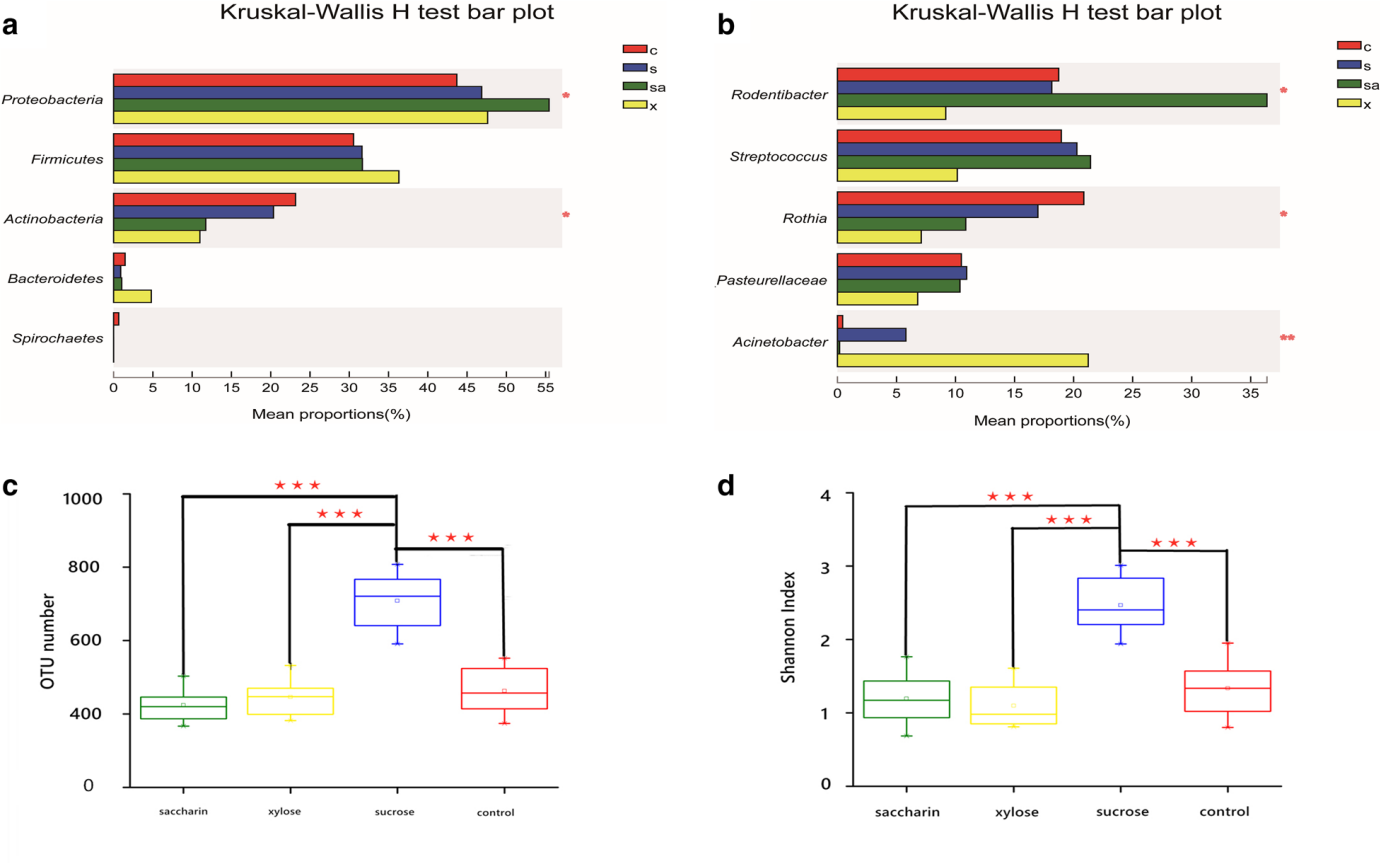
Statistical comparison of the relative abundance (**a**, **b**) and alpha diversities (**c**, **d**) of the oral microbiota observed in each of the four groups of rats. **a** Comparison of dominant phyla in the control (c group), xylose (x group), sucrose (s group), and saccharin (sa group) groups. **b** Comparison of the dominant genera in the control (c group), xylose (x group), sucrose (s group), and saccharin (sa group) groups. **c** Bacterial community richness in each of the four groups. **d** Bacterial community diversity in each of the four groups. *p* < 0.05 was considered significant. * 0.01 < *p* ≤ 0.05, ** 0.001 < *p* ≤ 0.01, *** *p* ≤ 0.001

A total of 284 genera were detected in the xylose group, and 186 genera were identified in the saccharin group, but the most diverse community was identified in the sucrose group samples which shared 372 genera. Multi-group comparison revealed that the relative abundance of the oral microbiota at the phylum level, which was dominated by *Proteobacteria* and *Actinobacteria*, was significantly different between these four groups (Fig. 2a). The relative abundance of *Rodentibacter, Streptococcus*, and *Rothia* was also demonstrated to be significantly different in the multigroup comparisons when evaluating the data at a genus level (Fig. 2b). The results of the pairwise comparisons can be found in Additional file 1: Figures S5–S7 and reflect our finding that the sucrose group exhibited the highest degree of bacterial community richness (measured as a function of the total number of observed OTUs) amongst the four groups (Fig. 2c, p < 0.005). Bacterial community diversity was also measured using the Shannon index and the sucrose group exhibited significantly higher diversity values when compared to the control, xylose, and saccharin groups (Fig. 2d, p < 0.005).

### Unique and shared bacterial taxa

**Fig. 3:**
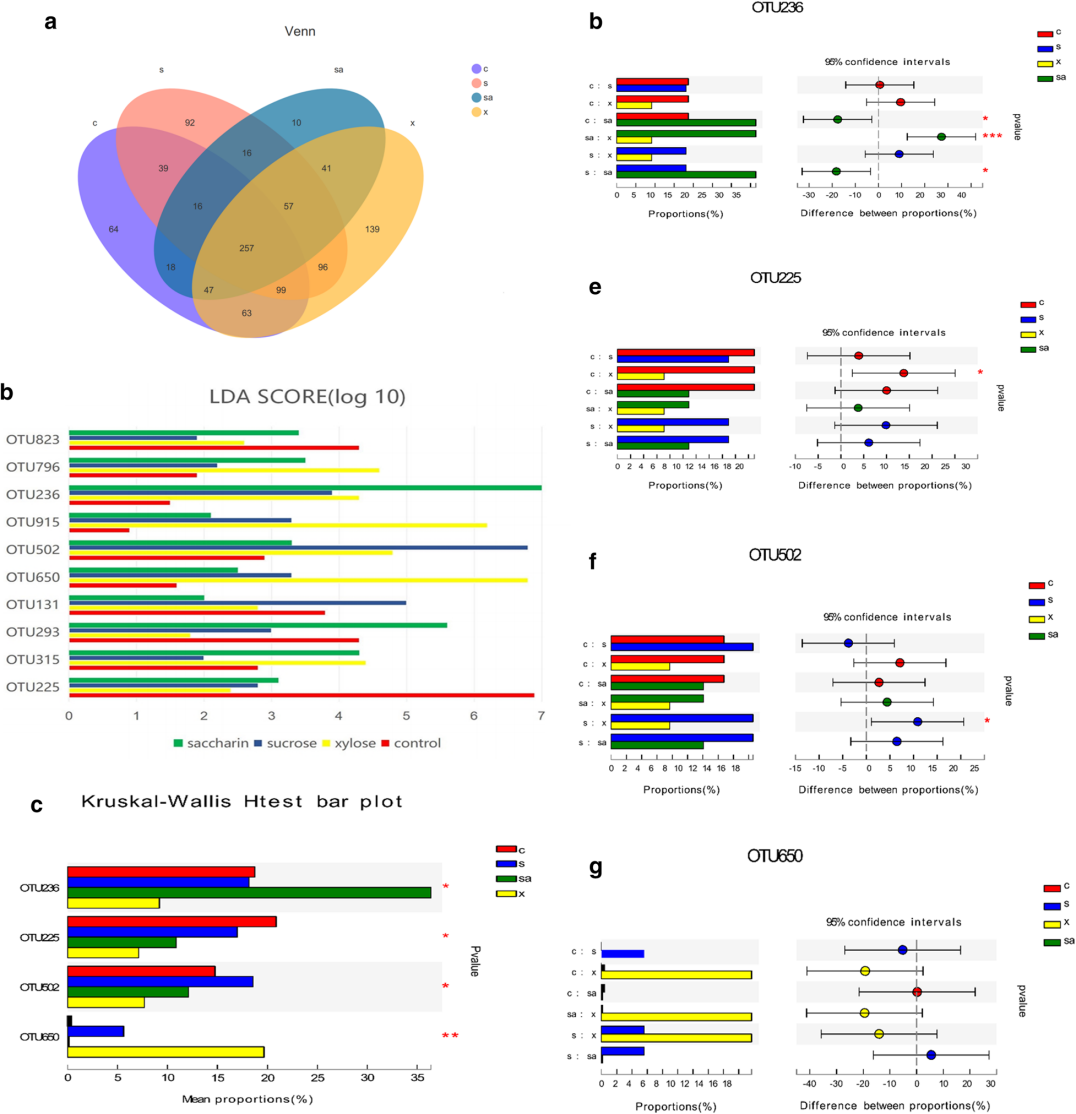
Unique and shared OTUs in each of the four treatment groups. **a** Venn diagram showing the shared OTUs (3% distance level) from each of the four groups. **b** Top ten differentially expressed OTUs identified in the control, xylose, saccharin and sucrose groups identified using linear discriminant analysis coupled with effect size (LEfSe). The distribution of the most differentially expressed OTUs in each group compared in pairs; OTU225, OTU502, OTU650 and OTU236 are illustrated in **c**, **d**, **e** and **f**, respectively. Four groups: control (c group, red color), xylose (x group, yellow color), sucrose (s group, blue color) and saccharin (sa group, green color) groups. * 0.01 < *p* ≤ 0.05, ** 0.001 < *p* ≤ 0.01, *** *p* ≤ 0.001

We then examined the unique and shared bacterial taxa between the oral microbiota of rats from the four treatment groups (xylose, saccharin, sucrose, and control) using our sequencing data. There were 257 shared OTUs in this dataset (Fig. 3a) and we identified differentially distributed OTUs among these treatment groups LEfSe. Among the 257 shared OTUs, the top ten OTUs were identified as 823, 796, 236, 915, 502, 650, 131, 293, 315, and 225 (Fig. 3b). Multi-group comparisons revealed that OTU225 (belonging to *Actinobacteria Rothia*), OTU236 (belonging to *Proteobacteria Rodentibacter*), OTU502 (belonging to *Firmicutes Streptococcus*), and OTU650 (belonging to *Proteobacteria Acinetobacter*) were significantly different between the four groups (Fig. 3c), while the Post-hoc tests revealed that OTU236 was most abundant in the saccharin group, which was significantly different from all the other groups (Fig. 3d). OTU225, was shown to be the most abundant in the control group which was significantly different from the xylose group (Fig. 3e). OTU502, was found to have the highest prevalence in the sucrose group, which was significantly different from the xylose group (Fig. 3f) and OTU650, was shown to be highly prevalent in the xylose group, but was not significantly enriched in this group when compared to the other groups (Fig. 3g).

### Functional predictions

**Fig. 4:**
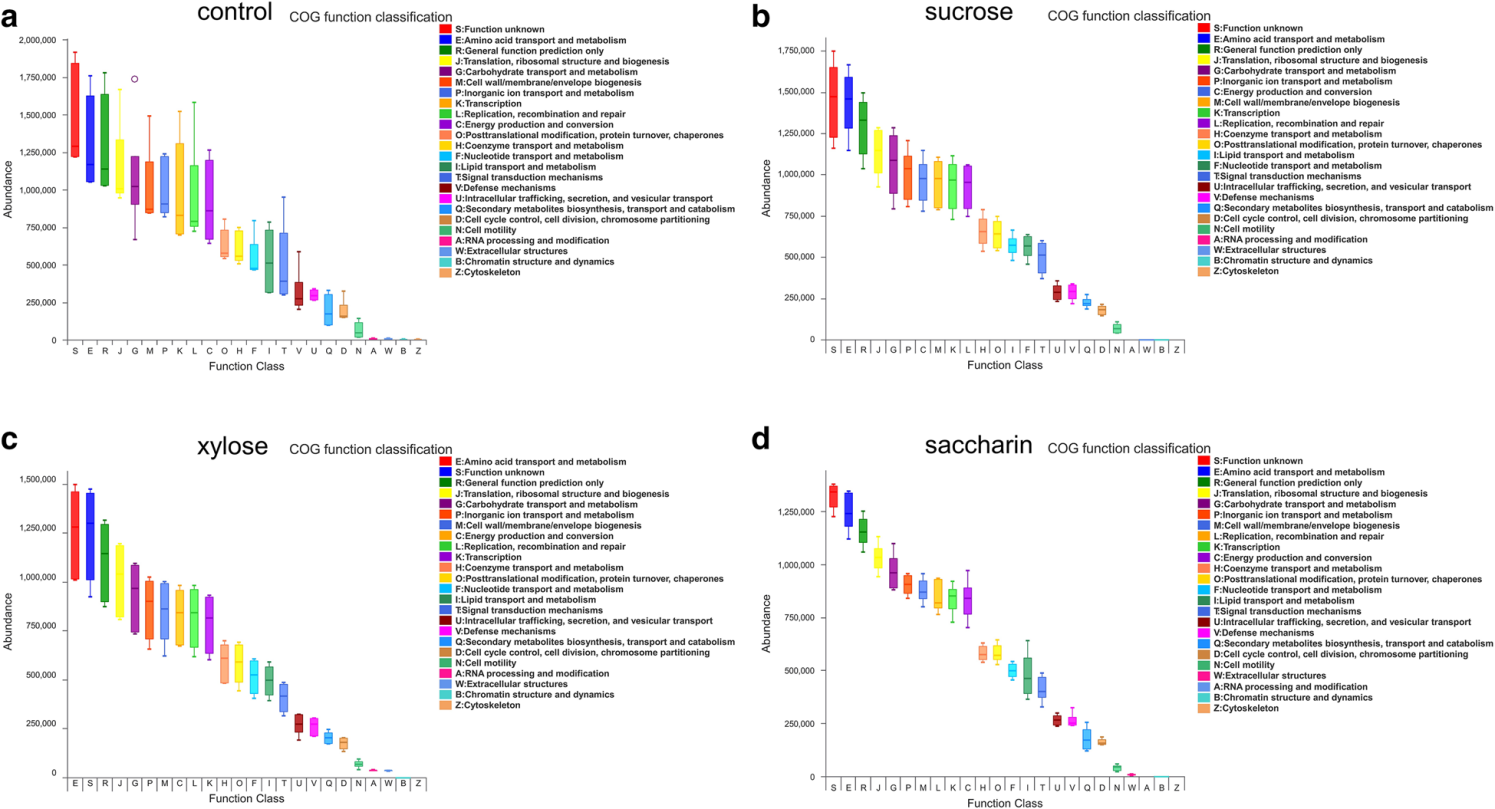
Box diagram describing COG functional classification statistics drawn based on the results of the PICRUSt functional prediction analysis

Based on the results of the 16S RNA sequencing, we mapped the most common microbial functions in each sample using PICRUSt metagenome prediction (Fig. 4). This analysis revealed that the functional profiles of the control, sucrose, and xylose group were extremely similar to each other, and showed no significant differences between each of these groups. Amino acid transport and metabolism, carbohydrate transport and metabolism, translation, and ribosomal structure and biogenesis were the most abundant functions associated with all four groups. However, there were a few functional differences in the saccharin group. For instance, the relative abundance of some of the functional profiles was decreased in the saccharin group compared to that in the other groups; these profiles included amino acid transport and metabolism, translation, ribosomal structure and biogenesis, carbohydrate transport and metabolism, inorganic transport and metabolism, cell wall/membrane/envelope biogenesis, replication, recombination and repair, transcription, and energy production and conversion.

## Discussion

Although xylose and saccharin have long been considered safe, research on the intestinal microbiome suggests that their long-term use may cause glucose intolerance and interfere with the body's energy homeostasis (Gerasimidis et al. 2020). In our study, we found that there were changes in the composition of the microbiome in animals treated with these compounds at the phylum level, with the abundance of *Proteobacteria* increasing significantly in the saccharin group, and that of *Actinobacteria* increasing in the xylose group compared with that in the control. Previous studies have shown that consumption of xylose and saccharin leads to the excessive growth of *Proteobacteria* in the mouse gut, resulting in the development of gut immune system disorders and eventually, systemic inflammation (Shin et al. 2015). Here we observed similar changes in the oral microbiota of the xylose group rats. The gut microbiota of obese individuals has been shown to consist of up to 20% more *Firmicutes* than that of lean individuals (Shin et al. 2015). Our evaluations at the genus level demonstrated that the abundance of *Rodentibacter* was significantly increased in the saccharin group compared with that in the other groups. Although *Rodentibacter* often colonize the human oral cavity, they are usually opportunistic in nature (Hurst et al. 2018). Additionally, the levels of *Staphylococcus*, *Acinetobacter*, and *Lactobacillus*—all species closely related to food fermentation—increased in the microbial samples from the xylose group. Fermentation is often accompanied by acid production, and excessive acid formation, which can damage the enamel of the teeth (Bretz et al. 2005). Dental caries typically begin at or below the enamel surface and are the result of a process where the crystalline mineral structure of the tooth is demineralized by organic acids produced by biofilm bacteria during dietary fermentation. *Staphylococcus*, *Acinetobacter*, and *Lactobacillus* are major biofilm producing bacteria, which are all able to produce lactic acid, the dominant end product of sugar metabolism and the primary acid involved in carie formation (Pitts et al. 2017).

PCoA revealed that the sucrose group was closely related to the control group. In contrast, the xylose and saccharin groups included significant changes to their composition and community structure compared to the control. This indicates that xylose and saccharin intake significantly change the oral microbiota composition, increasing the risk of oral micro-ecological imbalance. In addition, the sucrose group samples exhibited a significantly higher community diversity and species richness compared to other groups, which is consistent with previous reports that suggest that sucrose provides the metabolic substrates needed for many types of microbes to grow (Etxeberria et al. 2015). In summary, the relative abundance analysis suggests that all four groups present with variable and complex community structures with changes in both the composition and abundance of the colonizing microbes. Thus, long-term consumption of sucrose, xylose, and saccharin will change the oral micro-ecology balance, which may have a negative impact on the host.

The unique and shared bacterial taxa analysis indicated that the intake of sucrose, xylose, and saccharin interferes with the original micro-ecological balance of the oral cavity. For example, *Streptococcus*, which is often found in patients suffering from caries, is a dominant genus in the sucrose group. *Acinetobacter*, which is the primary pathogen in pneumonia, meningitis, peritonitis, endocarditis, urinary tract infections, and skin infections, was a core genus in the xylose group oral microbiome (Munoz-Price et al. 2008). In addition, PICRUSt analysis of the 16S rRNA sequencing data suggests that these treatments also alter the functional composition of the microbial communities. PICRUSt analysis indicated a decrease in the functional abundance of several metabolic functional features including amino acid and carbohydrate metabolism in the saccharin group, suggesting that the microbial metabolism in these rats may be repressed. However, no significant differences were observed among these functional profiles when compared at the individual level, which may be because the PICRUSt results were based on 16S rRNA sequencing, and further studies are needed to perform a more in depth metabolic analysis of these samples, before any making any conclusive statements regarding changes to their metabolism.

Changes in the oral immunoglobulin content may reflect the effects of sucrose, xylose, and saccharin on the oral immune system. In our experiment, although SIgA, IgG, and IgM were detected in all of the saliva samples, only SIgA and IgG were found to be highly expressed in the sucrose and xylose groups. IgM, which is primarily expressed following infection of the oral cavity was not shown to exhibit any significant changes in expression in any of the treatment groups and IgE was not detected in any of the samples, probably due to its low concentration in the saliva. SIgA and IgG are major components of saliva, gingival crevicular fluid, and other secretions in the oral cavity (El-Gebaly et.al. 2012). These results closely resemble the normal characteristics of these three immunoglobulins. However, patients with increased SIgA and IgG expression are more vulnerable to external influences (Brandtzaeg et al. 2007). Taken together our results suggest that both xylose and sucrose intake may affect the oral immune system. Although the sucrose and xylose groups demonstrated similar trends in their SIgA and IgG expression, the changes in their microbiomes were not consistent. No significant changes were observed in the sucrose group at the genus level when compared with the control while the xylose group demonstrated a significant change in the abundance of *Streptococcus*, *Rothia*, *Rodentibacter*, and *Enterobacter* compared to the control. To our surprise, even though the saccharin group did not demonstrate any significant changes in the expression of the oral immunoglobulins, the abundance of *Rodentibacter*, *Rothia*, *Staphylococcus*, and *Pseudomonas* was significantly different compared to that in the control. Whether these genera are associated with changes in immunoglobulin expression needs further analysis. However, changes in the oral immunoglobulin composition may reflect the effect of sweetener consumption on the oral immune system. This result is consistent with our previous microbiota composition analysis and suggests that they may indicate some underlying damage to the micro-ecological structures of the oral cavity.

In summary, our results reveal the effects of sucrose, xylose, and saccharin on the composition, diversity, and metabolic function of the oral microbiota. However, our study has certain limitations. For example, we have observed that these sweeteners interfered with the rat oral micro-environment and disrupted the oral micro-ecological balance, using only 24 samples. Thus, further studies using samples from larger study populations and human samples are required to validate our results. In addition, we did not measure the specific water intake, weight gain or water loss in these rats during the course of the entire study, which is another flaw in its design. More suitable animal models could provide more information on the effect of different sweeteners. Nevertheless, our results confirm the effect of different sweeteners on the oral microbiota and immune system, indicating the need to control the use of sucrose, xylose, and saccharin as beverage additives.

## Supplementary Information

**Additional file 1: Figure S1.** Rarefaction curves of oral microbiota in four groups rats. **Figure S2.** Shannon index of oral microbiota in four groups rats. **Figure S3.** Simpson index of oral microbiota in four groups rats. **Figure S4.** Coverage index of oral microbiota in four groups rats. **Table S1.** Four group rats diversity Index Table. **Figure S5.** Phylotypes significantly different between xylose and control groups at genus level. * 0.01 < *p *≤ 0.05, ** 0.001 < *p* ≤ 0.01, *** *p* ≤ 0.001. xylose vs. control group. **Figure S6.** Phylotypes significantly different between saccharin and control groups at genus level. * 0.01 < *p* ≤ 0.05, ** 0.001 < *p* ≤ 0.01, *** *p* ≤ 0.001. saccharin vs. control group. **Figure S7.** Phylotypes significantly different between sucrose and control groups at genus level. * 0.01 < *p* ≤ 0.05, ** 0.001 < *p* ≤ 0.01, *** *p* ≤ 0.001. sucrose vs. control group.

## Data Availability

All data were uploaded to the National Center for Biotechnology Information database (Accession Number: PRJNA607824).
