# Supplementary material for: Effect of different sweeteners on the oral microbiota and immune system of Sprague Dawley rats
Source: AMB Express. 2021 Jan 6;11:8. doi: 10.1186/s13568-020-01171-8 (PMC7788136; doi:10.1186/s13568-020-01171-8)
Supplement: Supplementary file 1 — Additional file 1: Figure S1. Rarefaction curves of oral microbiota in four groups rats. Figure S2. Shannon index of oral microbiota in four groups rats. Figure S3. Simpson index of oral microbiota in four groups rats. Figure S4. Coverage index of oral microbiota in four groups rats. Table S1. Four group rats diversity Index Table. Figure S5. Phylotypes significantly different between xylose and control groups at genus level. * 0.01 < p ≤ 0.05, ** 0.001 < p ≤ 0.01, *** p ≤ 0.001. xylose vs. control group. Figure S6. Phylotypes significantly different between saccharin and control groups at genus level. * 0.01 < p ≤ 0.05, ** 0.001 < p ≤ 0.01, *** p ≤ 0.001. saccharin vs. control group. Figure S7. Phylotypes significantly different between sucrose and control groups at genus level. * 0.01 < p ≤ 0.05, ** 0.001 < p ≤ 0.01, *** p ≤ 0.001. sucrose vs. control group. [file 13568_2020_1171_MOESM1_ESM.docx]

**Supplementary Materials:**

**Effect of different sweeteners on the oral microbiota and immune system of Sprague Dawley rats**

Xi Cheng^1，2^, Xiurong Guo^2^, Feihong Huang^2^, Hui Lei^2^, Quan Zhou^2^, Can Song^2^*

1.Department of Stomatology, People’s Hospital of Leshan, Leshan, 614000, Sichuan, China

2.School of Pharmacy, Southwest Medical University, Luzhou, 646000, Sichuan, China

*Corresponding: cansong@swmu.edu.cn (Can Song)


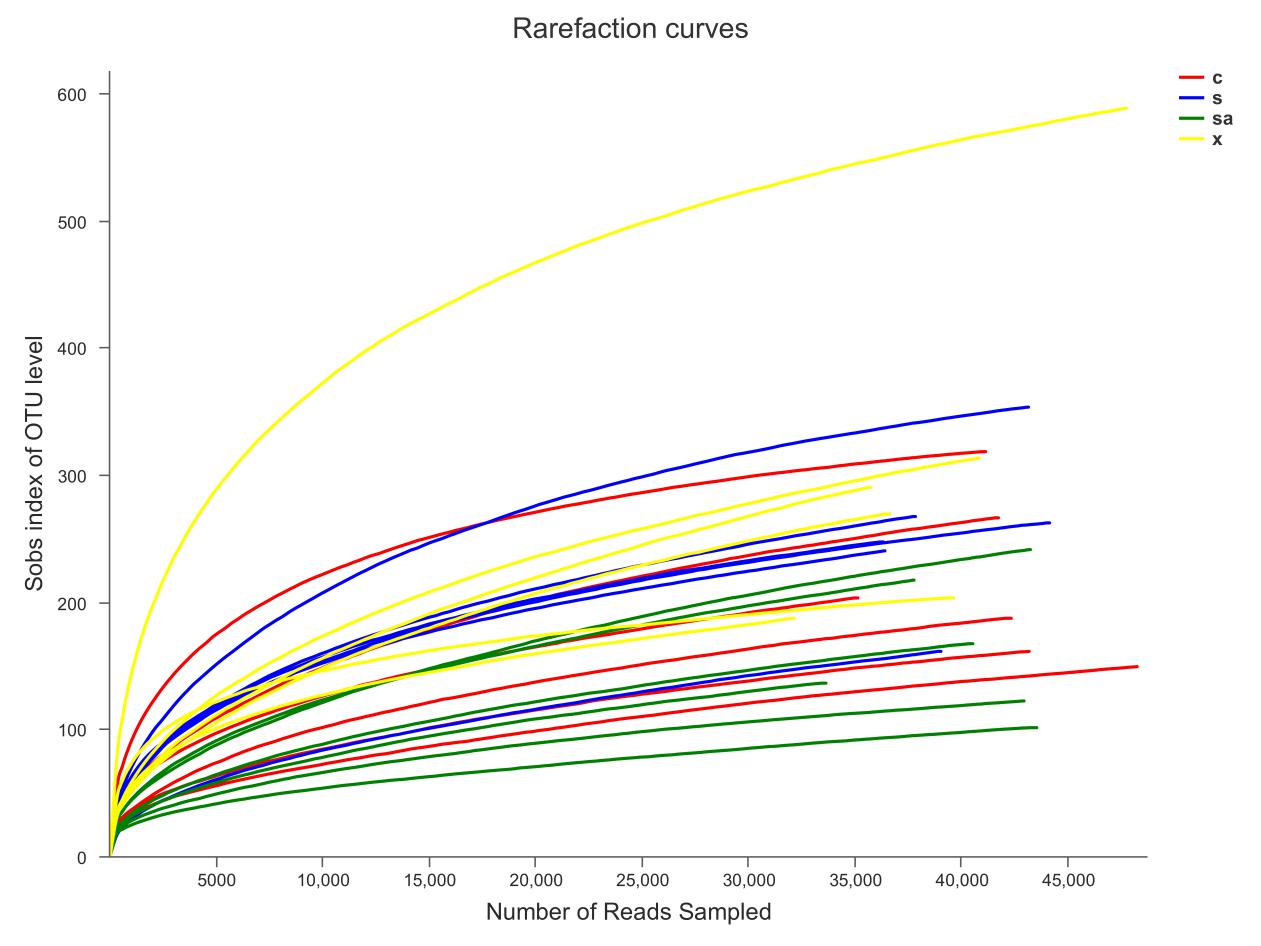


#### Figure.S1 Rarefaction curves of oral microbiota in four groups rats

####
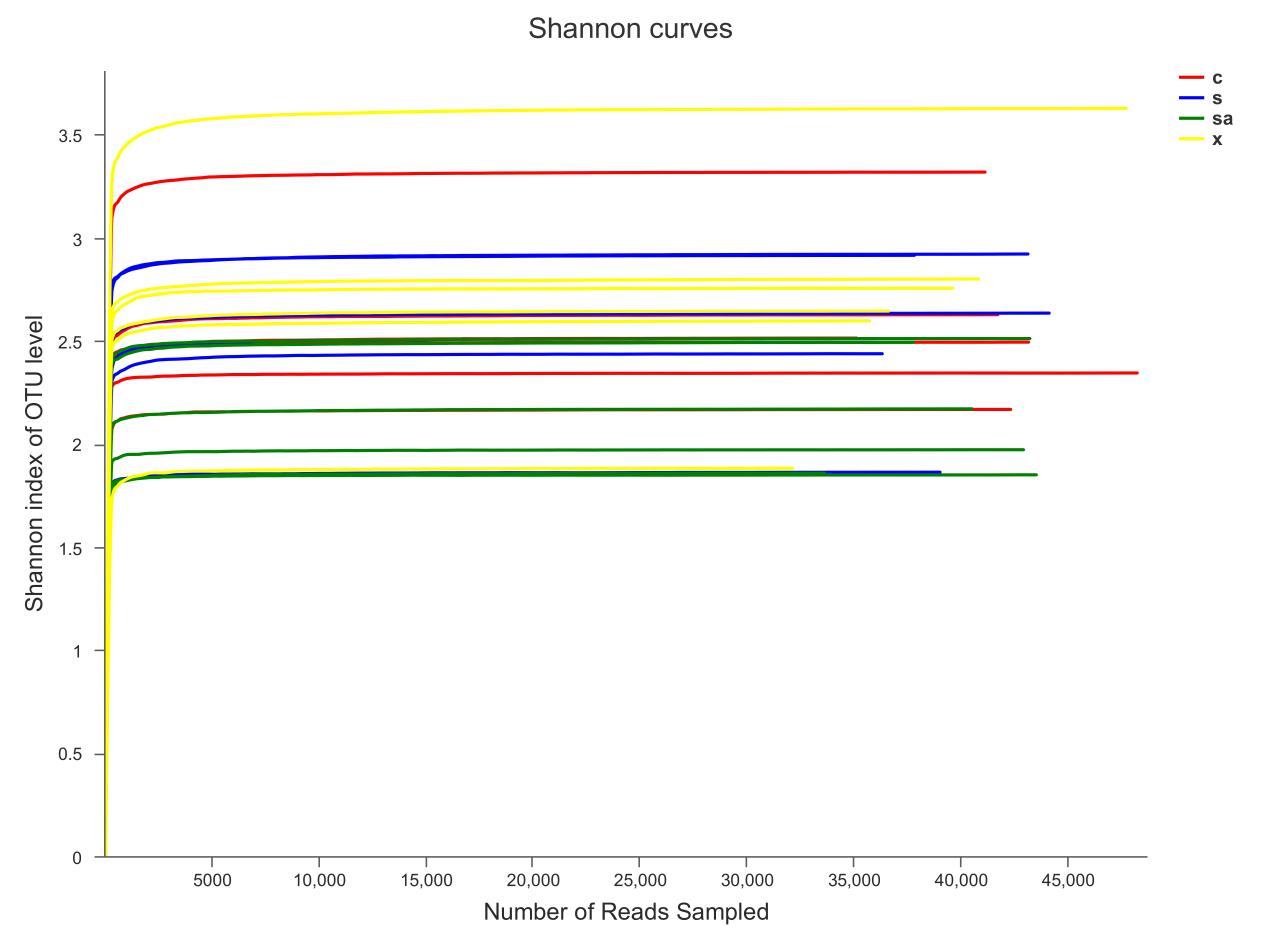


#### Figure.S2 Shannon index of oral microbiota in four groups rats


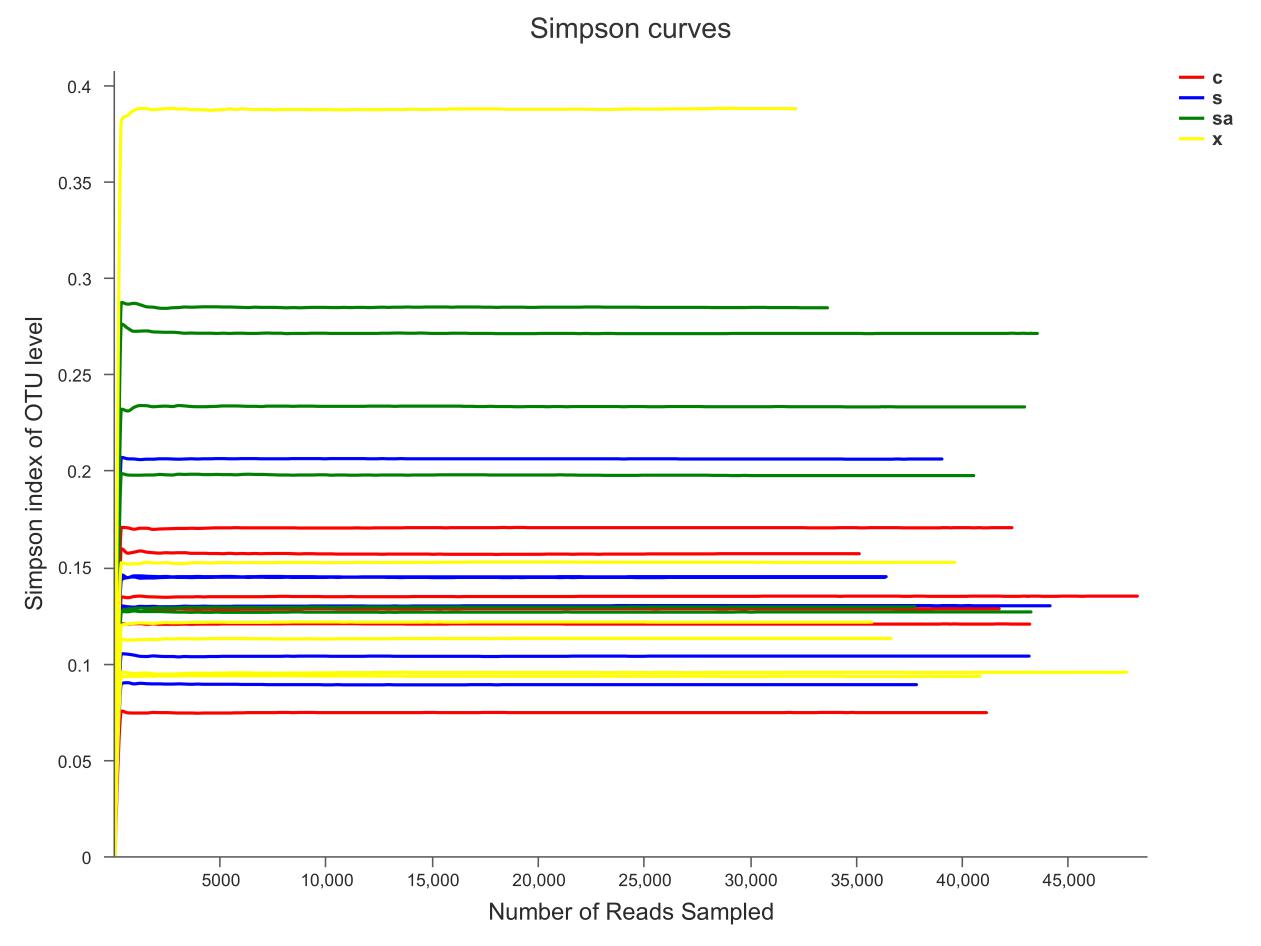


#### Figure.S3 Simpson index of oral microbiota in four groups rats


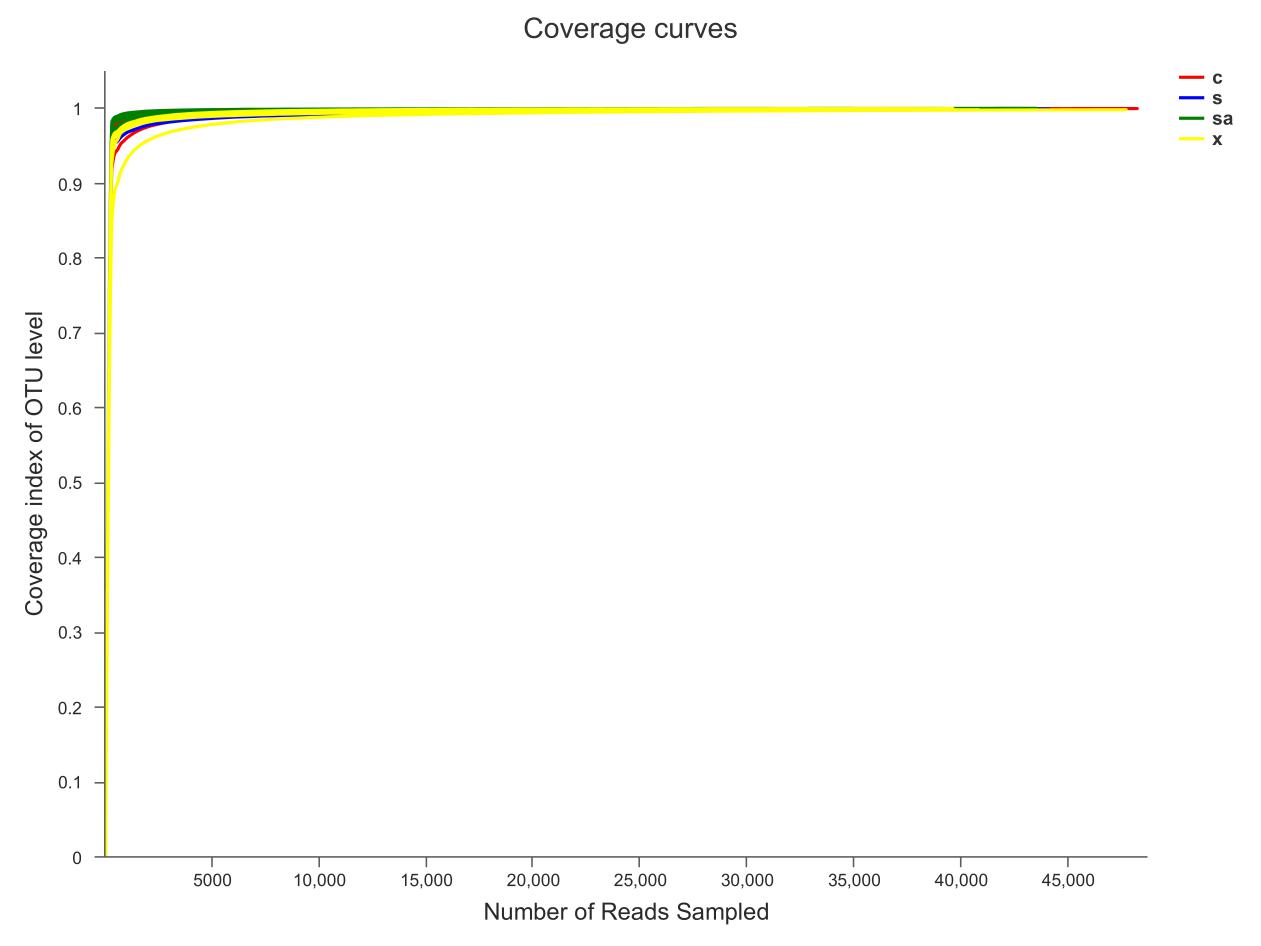


#### Figure.S4 Coverage index of oral microbiota in four groups rats

Table. S1 Four group rats diversity Index Table

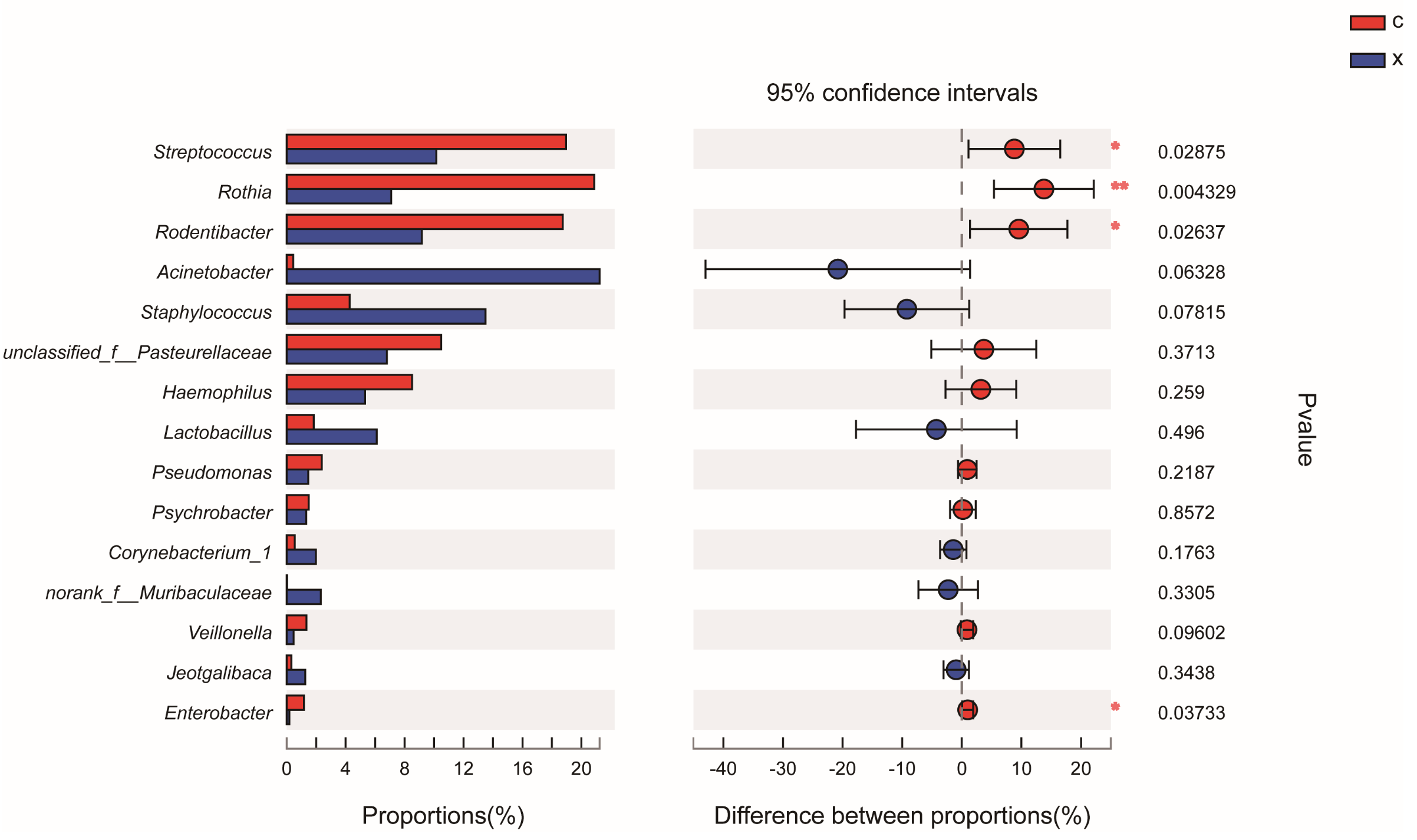


Figure.S5 Phylotypes significantly different between xylose and control groups at genus level.

* 0.01 < *p* ≤ 0.05，** 0.001 < *p* ≤ 0.01，*** *p* ≤ 0.001. xylose vs. control group.


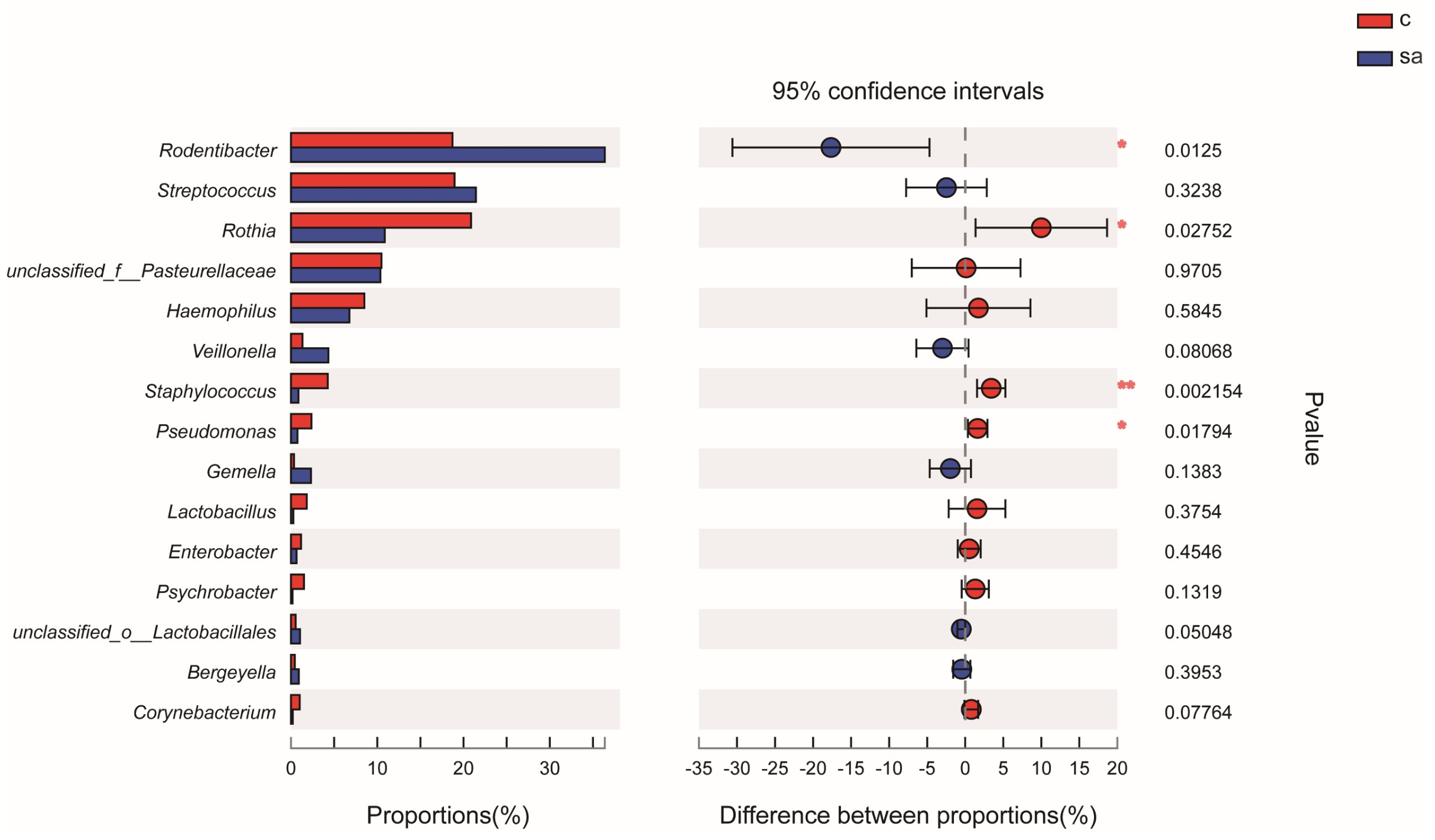


Figure.S6 Phylotypes significantly different between saccharin and control groups at genus level.

* 0.01 < *p* ≤ 0.05，** 0.001 < *p* ≤ 0.01，*** *p* ≤ 0.001. saccharin vs. control group.


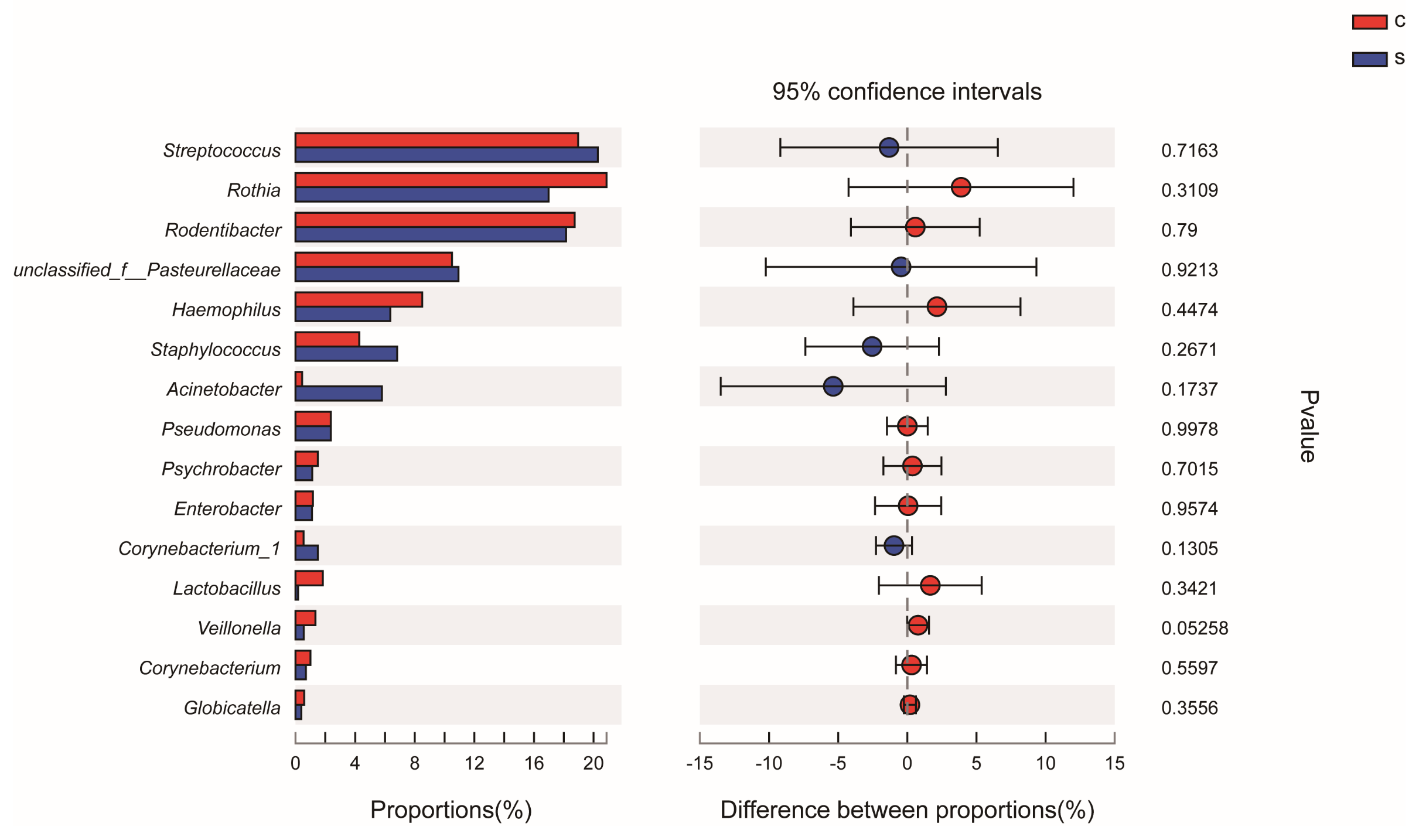


Figure.S7 Phylotypes significantly different between sucrose and control groups at genus level.

* 0.01 < *p* ≤ 0.05，** 0.001 < *p* ≤ 0.01，*** *p* ≤ 0.001. sucrose vs. control group.
